# Risk evaluation and incidence prediction of endolymphatic hydrops using multilayer perceptron in patients with audiovestibular symptoms

**DOI:** 10.1097/MD.0000000000041880

**Published:** 2025-03-14

**Authors:** Yun Hwa Chang, Ha Youn Kim, In Kyu Yu, Min Young Kwak

**Affiliations:** a Department of Radiology, Eulji University Hospital, Eulji University College of Medicine, Daejeon, Korea; b Department of Otolaryngology-Head and Neck Surgery, Eulji University Hospital, Eulji University School of Medicine, Daejeon, Korea.

**Keywords:** audiovestibular symptoms, endolymphatic hydrops, inner ear MRI, multilayer perceptron

## Abstract

Endolymphatic hydrops (EH) has been visualized on magnetic resonance imaging (MRI) in patients with various inner ear diseases. The purpose of this study was to evaluate the prevalence and risk factors of significant EH on inner ear MRI in patients with 1 or more audiovestibular symptoms and to predict the incidence of significant EH using multivariate analysis and multilayer perceptron artificial neural network modeling. This retrospective study included a total of 135 patients with 1 or more audiovestibular symptoms who do not meet the diagnostic criteria for MD and underwent inner ear MRI at our institution from July 2021 to January 2024. The EH grade of each patient was evaluated, and “significant EH” was considered grade II or III. Of 135 patients with 1 or more audiovestibular symptoms, 48 patients (35.6%) presented with significant EH and 87 patients (64.4%) without significant EH on inner ear MRI. The prevalence of significant EH was higher in males, which was statistically significant (*P *= .007). The prevalence of significant EH was higher in the right ear, and the mean age of patients with significant EH was 1.94 years higher, but no statistical significance was observed (*P* = .660 and .456, retrospectively). The odds ratio for significant EH development was 2.696 (95% confidence interval: 1.296–5.607) times higher in men, which was statistically significant. Predicting the incidence of significant EH development using multivariate analysis, sex was the only variable that was statistically significant (*P* = .008). Based on a predictive model using multilayer perceptron (MLP), the classification accuracy of the model was 79.5%. In our study, the male gender could be related to the risk of developing significant EH in patients with audiovestibular symptoms. The accuracy of our suggested MLP model for predicting the incidence of significant EH was 79.5%, with sex being the highest predictor importance. In the future, inner ear MRI and MLP neural network modeling can be combined as a noninvasive and precise support system in the diagnosis of EH.

## 1. Introduction

Endolymphatic hydrops (EH) is a pathological and anatomical condition characterized by distension of the endolymphatic compartment of the inner ear.^[1]^ EH primarily occurs in the cochlea and saccule of the vestibule but may progress to the utricle and semicircular canal.^[2]^ Although the specific mechanisms of EH development in a variety of diseases remain unknown, various conditions, including infection, trauma, tumors, and clinical disorders such as Meniere disease (MD), are reported to be associated with EH.^[3]^ Progressive hydrops is the result of permanent impairment of endolymph resorption, mainly caused by clinical disorders of the endolymphatic sac, including MD, labyrinthine syphilis, and delayed hydrops syndrome.^[4]^

The pathophysiology of EH is due to an increased endolymphatic volume caused by an imbalance between the secretion and resorption of the endolymph. This high endolymphatic volume causes distension of the structure bounding the endolymphatic space, which results in the bending of Reissner and basilar membranes and may rupture the boundary membranes.^[3]^ Moreover, previous studies have suggested that increased endolymphatic volume influences the ionic concentration of the endolymphatic fluid with potassium hyperpermeability, which is toxic to hair cells.^[5,6]^ Thus, EH progression results in clinical symptoms such as hearing loss and vertigo.

Visualization of the EH has been enabled with the use of delayed post-gadolinium (Gd) contrast sequences on magnetic resonance imaging (MRI). Since Gd contrast diffuses to the perilymph but not to the endolymph, the perilymph signal is altered, which allows differentiation between the 2 structures.^[7]^ Grading systems have been proposed to evaluate the EH severity on MRI and its association with the clinical parameters of MD.^[8,9]^ EH appears to be also visible in patients with various diseases, including idiopathic sudden sensorineural hearing loss (SNHL), viral infection, autoimmune diseases, and recurrent peripheral vestibulopathy.^[3,10,11]^ However, previous studies have shown that the EH MRI findings are associated with the progression of MD, and most studies have been performed on patients with MD.^[8,12,13]^ To the best of our knowledge, no prior study has examined the statistical significance of the association between sex and EH presence evaluated on MRI. Therefore, EH evaluation on inner ear MRI should be further examined in patients who present various audiovestibular symptoms that do not satisfy the criteria of the MD diagnostic guidelines and to find associations between sex, age, affected ear, and EH presence.

The purpose of this study was to evaluate the prevalence and risk factors of significant EH on inner ear MRI in patients with 1 or more audiovestibular symptoms and to predict the incidence of significant EH using multivariate analysis and multilayer perceptron (MLP) artificial neural network (ANN) modeling.

## 2. Materials and methods

### 2.1. Study population

Institutional review board approval was obtained (IRB No. EMC 2022-12-001-001). This retrospective study included 135 patients with 1 or more audiovestibular symptoms, including dizziness, tinnitus, ear fullness, and fluctuating hearing loss, but who did not meet the diagnostic criteria for MD and underwent inner ear MRI at our institution from July 2021 to January 2024.

### 2.2. Inner ear MRI acquisition

All MRI scans were performed using a 3-tesla MRI scanner (MAGNETOM® Skyra; Siemens Medical Solutions) with a 32-channel array head coil. All patients were administered a single dose (0.2 mL/kg body weight) of intravenous Gadobutrol (gadolinium-DO3A-butriol, Gadovist®) 4 hours prior to MRI scanning. The following sequences were acquired in all MRI examinations for evaluation of the inner ear anatomy and grading of EH: heavily T2-weighted (hT2W) magnetic resonance cisternography, hT2W 3-dimensional fluid-attenuated inversion recovery with an inversion time of 2250 ms (positive perilymph image), hT2W 3-dimensional inversion recovery with an inversion time of 2050 ms (positive endolymph image), and hybrid of reversed image of positive endolymph signal and native image of positive perilymph signal (HYDROPS). The hT2W magnetic resonance cisternography sequence was obtained as an anatomical reference of the total endolymphatic fluid images. The HYDROPS sequence was acquired by subtracting positive endolymph image from positive perilymph image to evaluate the degree of EH.^[12]^

### 2.3. Radiological evaluation of EH

Based on the grading systems introduced by Barath et al^[8]^ and Bernaerts et al,^[9]^ EH was graded by an experienced neuroradiologist and a 3rd-year radiology resident. EH in the vestibule and cochlea was independently evaluated by comparing the relative areas of the contrast-enhanced perilymph space to the nonenhanced endolymphatic space on axial HYDROPS images.

Cochlear hydrops was evaluated according to the criteria introduced by Barath et al,^[8]^ in which the degree of hydrops is categorized as normal, grade I, or grade II. In the normal cochlea, the scala tympani, scala vestibuli, and interscalar septum are individually recognized. In grade I cochlear hydrops, the scala media becomes ambiguous, and the dark signal intensity replaces the structure owing to mild dilation of the nonenhancing cochlear duct. In grade II cochlear hydrops, the cochlear duct is further distended, and the scala vestibuli are entirely obliterated as the hydrops progresses.

Vestibular hydrops was evaluated based on a modified 4-stage grading system introduced by Bernaerts et al,^[9]^ in which the degree of hydrops is categorized as normal, grades I, II, and III. The saccule and utricle are recognized separately in the normal vestibule. In grade I vestibular hydrops, the saccule, the smallest component of the vestibular sacs, becomes equal to or larger than the utricle; however, the saccule and utricle are still separately recognized. In grade II vestibular hydrops, the saccule and utricle are fused, but an enhanced perilymphatic space is still present. In grade III vestibular hydrops, peripheral rim enhancement of the perilymphatic space is no longer visible, and the bony vestibule becomes fully obliterated.

In this study, the EH grade of each patient was evaluated, and “significant EH” was considered as grade II cochlear hydrops and grade II or III vestibular hydrops since mild EH (grade I) was clinically equivocal. Previous research showed that as the diagnostic scale of MD increased in the order unaffected, possible, probable, and definite, the proportions of severe hydrops increased. Moreover, the presence and severity of EH was significantly increased in the clinically affected ear compared to the unaffected ear.^[8,14]^

### 2.4. Statistical analyses

Statistical data analyses were performed with IBM SPSS Statistics 28.0 (SPSS Inc., Chicago, IL). The general characteristics of the study population were calculated as mean (standard deviation) and frequency (%). The comparisons within the groups with significant EH were performed using the Mann-Whitney test and Chi-square normality test after normality verification. For risk evaluation of developing significant EH, the odds ratio (OR) was analyzed based on sex and the site of symptomatic ear (left or right). In addition, multivariate analysis and MLP ANN modeling analysis were performed to predict the incidence of significant EH. *P* values < .05 were considered statistically significant.

### 2.5. ANN modeling analysis

**Figure 1.**
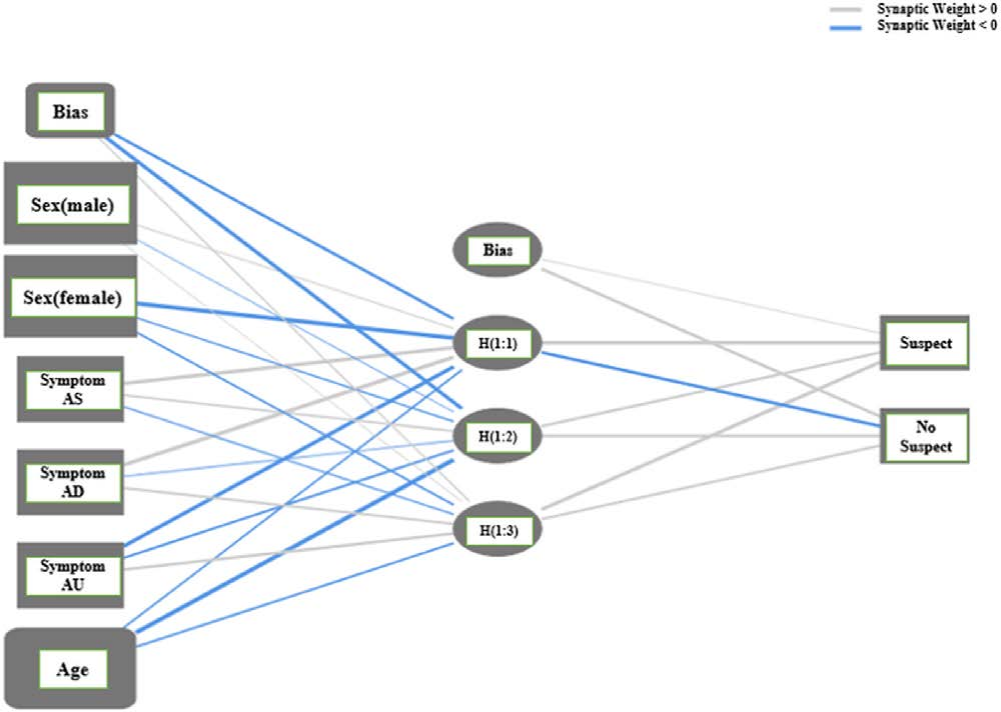
Multilayer perceptron model. A multilayer perceptron model consists of an input layer, 1 hidden layer, and an output layer. An input layer consists of the independent variables: sex (male and female), age, and the site of the symptomatic ear (left, right, and both). The hidden layer network is consisted of 1 hidden layer, 3 nodes, and a hyperbolic tangent activation function. The output layer network consists of a dependent variable, 2 nodes, a softmax activation function, and a cross-entropy loss function. The dependent variable is the incidence of significant endolymphatic hydrops. The neurons in each layer are structured to be completely connected to the previous layer. AD = right, AS = left, AU = both, Suspect = significant.

MLP ANN modeling analysis was performed to predict the incidence of significant EH (Fig. 1). MLP is a form of ANN consisting of an input layer, 1 or more hidden layers, and an output layer. The neurons in each layer are structured to be completely connected to the previous layer. The hidden layer network consisted of 1 hidden layer, 3 nodes, and a hyperbolic tangent activation function. The output layer network consisted of a dependent variable, 2 nodes, a softmax activation function, and a cross-entropy loss function. The MLP was modeled with training and testing sets (5:5). The independent variables used in the model were sex, age, and the site of symptomatic ear, and the dependent variable was the incidence of significant EH.

## 3. Results

### 3.1. Demographics

This retrospective study included a total of 135 patients (50 males and 85 females) with 1 or more audiovestibular symptoms, including dizziness, tinnitus, ear fullness, and fluctuating hearing loss. The mean age was 48.3 years, ranging from 13 to 81 years.

### 3.2. Imaging findings

**Figure 2.**
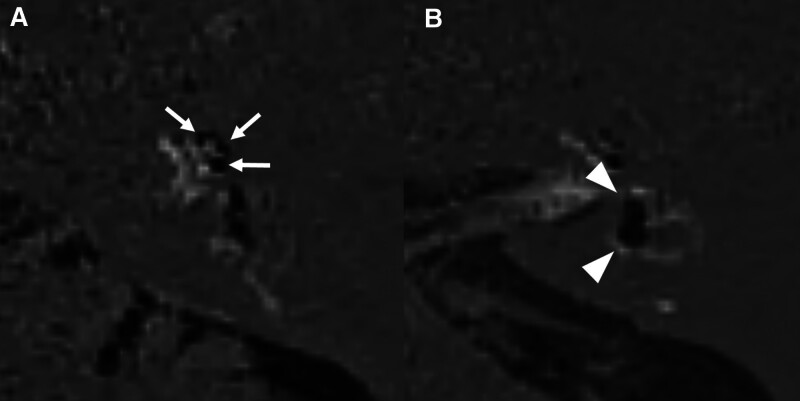
Inner ear magnetic resonance imaging (MRI) of a 60-year-old male with dizziness and left tinnitus. (A) Grade II cochlear hydrops is detected on a 0.1 mm axial hybrid of reversed image of positive endolymph signal and native image of positive perilymph signal (HYDROPS) image of inner ear MRI at cochlear level, in which a nodular black cutout of the scala vestibuli (arrows) is visible. (B) Grade II vestibular hydrops is detected on a 0.1 mm axial HYDROPS image at vestibular level, in which a complete confluence of saccule and utricle (arrowheads), occupying almost all the vestibule, is visible, but with visible enhancement of the perilymphatic rim.

**Figure 3.**
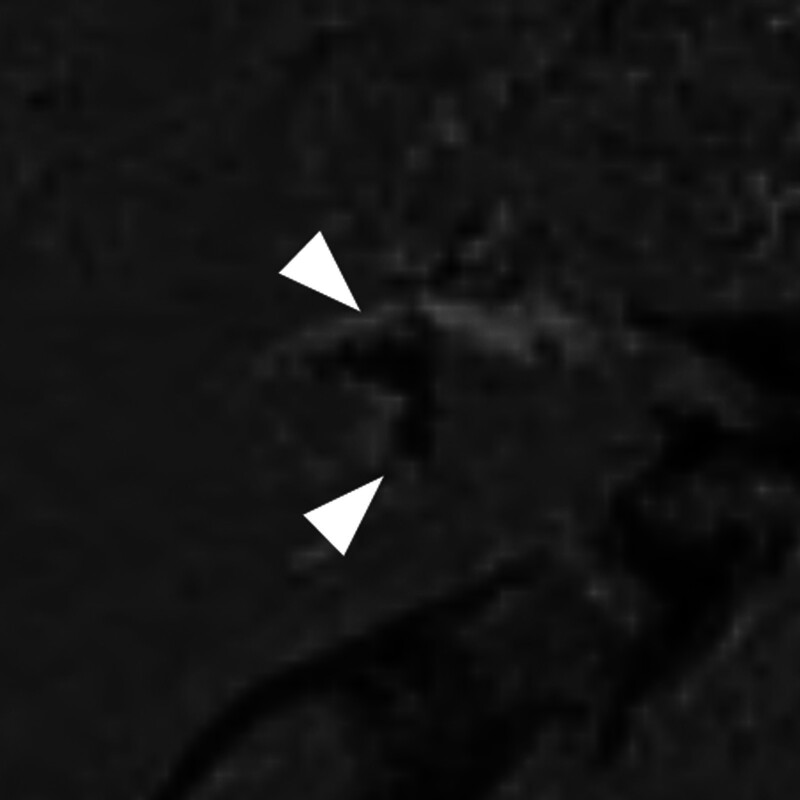
Inner ear magnetic resonance imaging (MRI) of a 46-year-old female with dizziness and right ear fullness. Grade II vestibular hydrops is detected on a 0.1 mm axial hybrid of reversed image of positive endolymph signal and native image of positive perilymph signal (HYDROPS) image of inner ear MRI at vestibular level, in which a complete confluence of saccule and utricle (arrowheads) with enhancement of the perilymphatic rim is visible.

**Figure 4.**
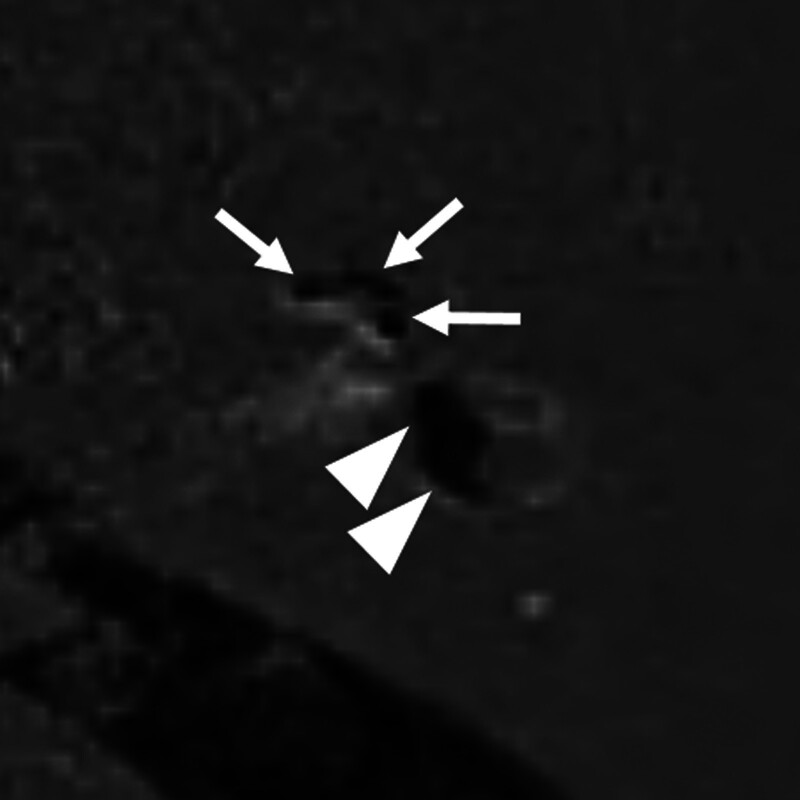
Inner ear magnetic resonance imaging (MRI) of a 45-year-old male with left sudden sensorineural hearing loss. Grade II cochlear hydrops and grade III vestibular hydrops are detected on a 0.1 mm axial hybrid of reversed image of positive endolymph signal and native image of positive perilymph signal (HYDROPS) image of inner ear MRI, in which a fully obliterated scala vestibuli (arrows) and a complete confluence of saccule and utricle (arrowheads) without a distinct enhancing perilymphatic rim are visible.

Significant EH on inner ear MRI was observed in 48 (35.6%) of 135 patients with 1 or more audiovestibular symptoms, and no significant EH was observed in 87 patients (64.4%). Different degrees of EH on axial HYDROPS images in patients with audiovestibular symptoms are presented in Figures 2 to 4. Significant vestibular (grade II) and cochlear EH (grade II) were detected in a 60-year-old male with dizziness and left tinnitus (Fig. 2). Significant vestibular EH (grade II) without cochlear EH was visible in a 46-year-old female with dizziness and right ear fullness (Fig. 3). Significant hydrops in both cochlea (grade II) and vestibule (grade III) were shown in a 45-year-old male with left sudden sensorineural hearing loss (Fig. 4).

### 3.3. General characteristics and significant EH

General characteristics of the study population are shown in Table 1. There was a statistically significant (*P* = .007) higher prevalence of significant EH in men in the group with significant EH, which consisted of 25 men (52.1%) and 23 women (47.9%), compared to the group without significant EH, which consisted of 25 men (28.7%) and 62 women (71.3%). The group with significant EH had a mean age of 49.33 ± 14.50 years, while the group without significant EH had a mean age of 47.39 ± 15.29 years. The significant EH group had a higher mean age of 1.94 years (*P* = .456). The left ear was clinically affected in 21 patients (43.8%), the right ear in 23 patients (47.9%), and both ears in 4 patients (8.3%) in the group with significant EH. In the group without significant EH, the left ear was clinically affected in 45 patients (51.7%), the right ear in 35 patients (40.2%), and both ears in 7 patients (8.0%). Although the right ear had a higher prevalence of significant EH visible on inner ear MRI, there was no statistically significant difference (*P* = .660).

**Table 1:** General characteristics of the study population with and without significant endolymphatic hydrops.

		Endolymphatic hydrops				*X*^2^/Z	*P* value
		Significant		No significant			
		N/M	%/SD	N/M	%/SD		
Sex	Male	25	52.1	25	28.7	7.231	.007
	Female	23	47.9	62	71.3		
Age		49.33	14.50	47.39	15.29	−0.745	.456
Affected ear	Left	21	43.8	45	51.7	0.831	.660
	Right	23	47.9	35	40.2		
	Both	4	8.3	7	8.0		

Z = Mann-Whitney test, *X*^*2*^ = Chi-square test, *P*-value < .05.

### 3.4. Risk evaluation of significant EH

The OR and 95% confidence intervals (CI) of developing significant EH according to sex and symptomatic ear are shown in Table 2. According to sex, men had a 2.696 (95% CI: 1.296–5.607) times greater OR of developing significant EH. The 95% CI range did not encompass 1, which was statistically significant. The OR was 1.408 (95% CI: 0.673–2.946) times greater in the right ear than in the left ear, but as the 95% CI range included 1, it was not statistically significant.

**Table 2:** Risk estimates of significant endolymphatic hydrops according to sex and symptomatic ear.

Odds ratio	Value	95% CI	
		Lower	Upper
Sex*	2.696	1.296	5.607
Symptomatic ear†	1.408	0.673	2.946

CI = confidence interval.

* Sex (male/female).

† Symptomatic ear (left/right).

### 3.5. Incidence prediction of significant EH

Multivariate analysis, a widely utilized statistical method, was used to predict the incidence of significant EH development, as seen in Table 3. The Hosmer-Lemeshow test revealed a good fit for the model with *X*^2^ = 1.133 (*P* = .997). In comparison to men, women had a 0.370 (95% CI: 0.177–0.774) times reduced incidence risk of developing significant EH. A slightly greater incidence risk of developing significant EH was associated with age 1.007 [95% CI: 0.983–1.033]). The incidence risk of significant EH development was 1.199 (95% CI: 0.302–4.755) times greater in both ears and 1.468 (95% CI: 0.685–3.145) times in the right ear, compared to the left. Nevertheless, in the final multivariate analysis, sex was the only variable that was statistically significant (*P* = .008).

**Table 3:** Prediction of significant endolymphatic hydrops development using multivariate analysis.

Variables	Exp(B)	95% CI		*P* value
		LLCI	ULCI	
Sex (female)	0.370	0.177	0.774	.008
Age, yr	1.007	0.983	1.033	.563
Symptomatic right	1.468	0.685	3.145	.324
Symptomatic left	1.199	0.302	4.755	.797

CI = confidence interval, LLCI = lower level confidence interval, ULCI = upper level confidence interval.

*P*-value < .05.

**Figure 5.**
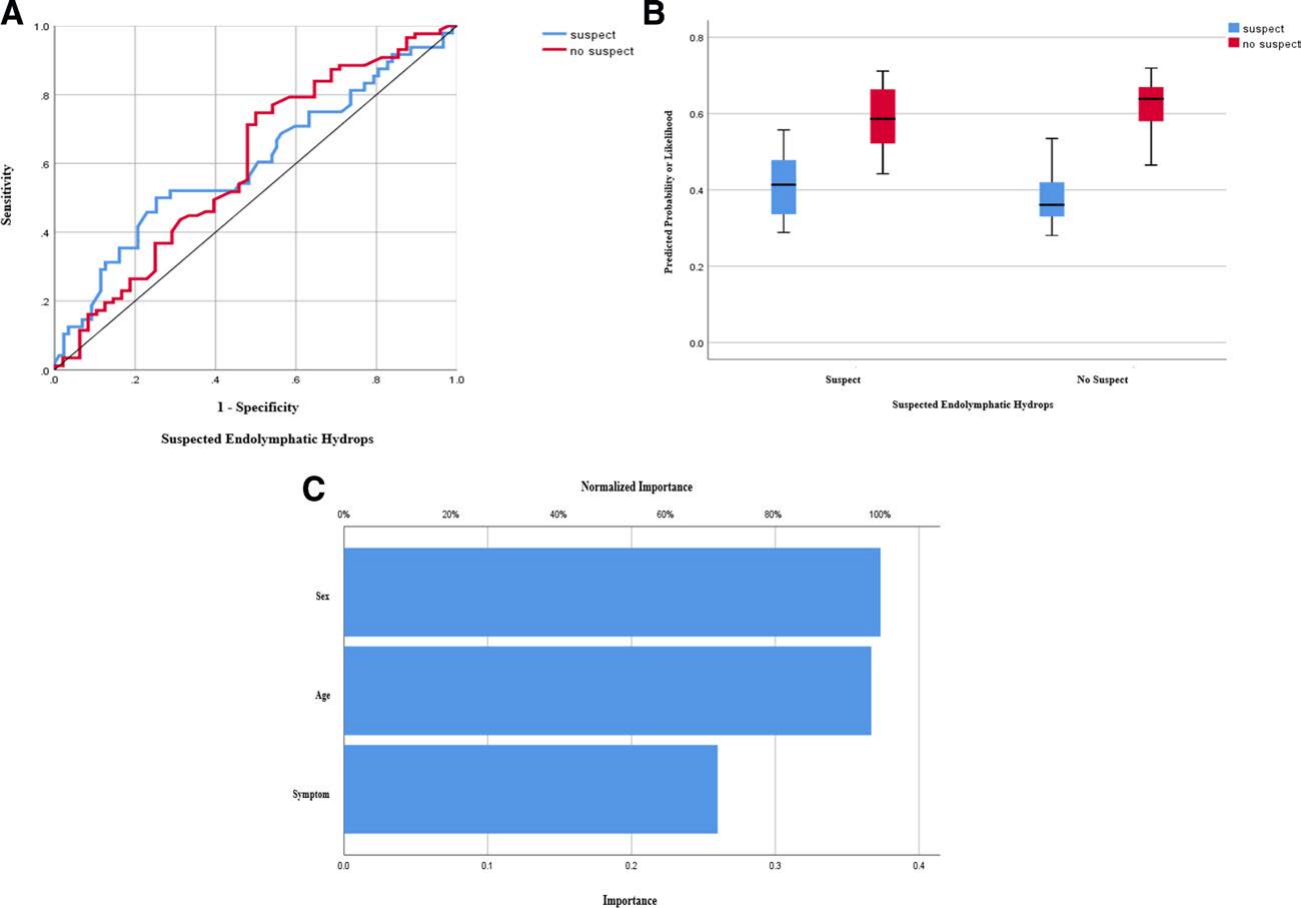
Multilayer perceptron output. (A and B) Our multilayer perceptron (MLP) model’s accuracy for predicting the incidence of significant endolymphatic hydrops was 79.5%. (C) Our MLP model output presents the predictor importance, sex being the highest predictor.

Using artificial intelligence (AI), an MLP predictive model was developed to predict the incidence of significant EH, as shown in Figure 5. The model’s classification accuracy was 79.5%. The predictor importance was 37.3% for sex, 36.6% for age, and 26.0% for the site of symptomatic ear, respectively.

## 4. Discussion

EH is a pathological and anatomical feature in which the endolymphatic compartment of the inner ear is distended by increased endolymphatic volume. It is considered a characteristic pathological finding of MD.^[14]^ Using delayed post-Gd contrast sequences, EH has been visualized on in vivo inner ear MRI, and its severity has been assessed in relation to the clinical parameters of MD. Since EH is recognized as the histopathological hallmark of MD, the majority of earlier research has been conducted on patients with definite MD.

Inner ear MRI has been increasingly applied recently to support the diagnosis of MD and to evaluate the disease progression.^[13,15]^ However, EH has also been detected in patients with a variety of different otological disorders, such as SNHL, inflammation, and recurrent vestibulopathy, which result in various audiovestibular symptoms, including dizziness, tinnitus, and fluctuating hearing loss.^[16]^

In this study, the EH grade of the cochlea and vestibule on inner ear MRI was assessed in 135 patients with 1 or more audiovestibular symptoms who do not meet the diagnostic criteria for MD. Among 135 patients, significant EH was found in 48 patients (35.6%). Previous research by Naganawa and Nakashima^[1]^ also demonstrated that while all patients with MD had EH visualized on inner ear MRI, not all subjects with detectable EH on MRI presented the classic MD symptoms. Furthermore, Domínguez et al found that there were significant differences between the groups, with a higher OR for EH presence in the definite MD group than in all other patients with audiovestibular symptoms who do not fulfill the diagnostic criteria for definite MD. The study included a total of 170 patients: 83 with definite MD, 38 with fluctuating SNHL, 34 with recurrent vertigo, and 15 with idiopathic sudden SNHL.^[17]^ In fluctuating SNHL and recurrent vertigo groups, 7 (20%) and 6 (16%) patients, respectively, were found to have severe cochlear and/or severe vestibular EH.^[17]^ The incidence of severe EH was slightly higher in patients without MD than in previous results. However, our study’s proportion of significant EH presence in patients with various audiovestibular symptoms was lower than the percentages previously reported in definite MD patients, as consistent with the previous report.

In our study, the prevalence of significant EH was significantly higher in males (*P *= .007). Moreover, the risk of developing significant EH was increased significantly in males (OR: 2.696 [95% CI: 1.296–5.607]). Prior research on the existence of EH visualized on MRI in MD patients has indicated a minor female preponderance.^[18,19]^

The group with significant EH had a higher mean age (49.33 ± 14.50 years) than the group without significant EH (47.39 ± 15.29 years), but it was not statistically significant. In a previous study, Maeda et al^[20]^ demonstrated that the development of EH in neurotologic patients was not correlated with chronological age. Dieterich et al^[21]^ showed a positive correlation between age and the endolymphatic space, in which the age-dependent changes in the endolymphatic space in participants with normal vestibulocochlear testing were examined.

In this study, the incidence prediction of significant EH was performed using an MLP neural network model of AI techniques. In earlier research, AI techniques have been used on the EH presence on inner ear MRI to evaluate EH ratios by a fully automated analytic system.^[22,23]^ Furthermore, recent research investigated the diagnostic performance of a machine learning model in identifying MD patients based on radiomic features extracted from conventional MRI scans.^[24]^ However, no research has investigated the incidence prediction regarding EH development using AI techniques. Our suggested MLP model’s accuracy for predicting the incidence of significant EH was 79.5%, with predictor importance of 37.3% for sex, 36.6% for age, and 26.0% for the site of symptomatic ear, respectively.

Although it was not statistically significant, we also found that the prevalence of EH was higher in the right ear.

This study has several limitations. First, the number of patients included in this study was relatively small. Future studies should be conducted with a larger number of patients. Second, selection bias may exist; since inner ear MRI was performed in patients with 1 or more audiovestibular symptoms, the incidence of significant EH could have been higher than in the inner ear syndrome patients.

## 5. Conclusion

In our study, the prevalence of significant EH was significantly associated with sex. Furthermore, there was a notable rise in the male’s risk of significant EH development. The accuracy of our suggested MLP model for predicting the incidence of significant EH was 79.5%, with sex being the highest predictor importance. Thus, inner ear MRI for EH evaluation in patients with various audiovestibular symptoms is recommended, especially for male and older patients, to assess the disease severity and its progression. In the future, inner ear MRI and MLP neural network can be combined as a noninvasive and precise support system in the diagnosis of EH.

## Author contributions

**Data curation:** Yun Hwa Chang, Ha Youn Kim.

**Formal analysis:** Yun Hwa Chang, Ha Youn Kim.

**Visualization:** Yun Hwa Chang.

**Writing—original draft:** Yun Hwa Chang.

**Writing—review & editing:** Yun Hwa Chang, Ha Youn Kim, In Kyu Yu, Min Young Kwak.

**Conceptualization:** Ha Youn Kim, In Kyu Yu, Min Young Kwak.

**Methodology:** Ha Youn Kim.

**Supervision:** Ha Youn Kim, In Kyu Yu.

**Validation:** Ha Youn Kim.

## References

[1] Naganawa S Nakashima T. Visualization of endolymphatic hydrops with MR imaging in patients with Ménière’s disease and related pathologies: current status of its methods and clinical significance. Jpn J Radiol. 2014;32:191–204. 24500139 10.1007/s11604-014-0290-4

[2] Conte G Lo Russo FM Calloni SF. MR imaging of endolymphatic hydrops in Ménière’s disease: not all that glitters is gold. Acta Otorhinolaryngol Ital. 2018;38:369–76. 30197428 10.14639/0392-100X-1986 PMC6146579

[3] Ferster APO Cureoglu S Keskin N Paparella MM Isildak H. Secondary endolymphatic hydrops. Otol Neurotol. 2017;38:774–9. 28306649 10.1097/MAO.0000000000001377 PMC5425947

[4] Schuknecht HF. Pathophysiology of endolymphatic hydrops. Arch Otorhinolaryngol. 1976;212:253–62. 1086664 10.1007/BF00453673

[5] Takeda T Takeda S Kakigi A. A possible mechanism of the formation of endolymphatic hydrops and its associated inner ear disorders. Auris Nasus Larynx. 2020;47:25–41. 31623941 10.1016/j.anl.2019.09.005

[6] Salt AN Plontke SK. Endolymphatic hydrops: pathophysiology and experimental models. Otolaryngol Clin North Am. 2010;43:971–83. 20713237 10.1016/j.otc.2010.05.007 PMC2923478

[7] Naganawa S Satake H Kawamura M Fukatsu H Sone M Nakashima T. Separate visualization of endolymphatic space, perilymphatic space and bone by a single pulse sequence; 3D-inversion recovery imaging utilizing real reconstruction after intratympanic Gd-DTPA administration at 3 Tesla. Eur Radiol. 2008;18:920–4. 18324405 10.1007/s00330-008-0854-8

[8] Baráth K Schuknecht B Naldi AM Schrepfer T Bockisch CJ Hegemann SCA. Detection and grading of endolymphatic hydrops in Menière disease using MR imaging. AJNR Am J Neuroradiol. 2014;35:1387–92. 24524921 10.3174/ajnr.A3856 PMC7966587

[9] Bernaerts A Vanspauwen R Blaivie C. The value of four stage vestibular hydrops grading and asymmetric perilymphatic enhancement in the diagnosis of Menière’s disease on MRI. Neuroradiology. 2019;61:421–9. 30719545 10.1007/s00234-019-02155-7 PMC6431299

[10] Chen X Zhang XD Gu X Fang ZM Zhang R. Endolymphatic space imaging in idiopathic sudden sensorineural hearing loss with vertigo. Laryngoscope. 2012;122:2265–8. 22996668 10.1002/lary.23452

[11] Attye A Dumas G Tropres I. Recurrent peripheral vestibulopathy: is MRI useful for the diagnosis of endolymphatic hydrops in clinical practice? Eur Radiol. 2015;25:3043–9. 25820480 10.1007/s00330-015-3712-5

[12] Cho YS Ahn JM Choi JE. Usefulness of intravenous gadolinium inner ear MR imaging in diagnosis of Meniere’s disease. Sci Rep. 2018;8:17562. 30510158 10.1038/s41598-018-35709-5 PMC6277445

[13] Connor SEJ Pai I. Endolymphatic hydrops magnetic resonance imaging in Meniere’s disease. Clin Radiol. 2021;76:76.e1–76.e19. 10.1016/j.crad.2020.07.021 32892985

[14] Han SC Kim YS Kim Y. Correlation of clinical parameters with endolymphatic hydrops on MRI in Meniere’s disease. Front Neurol. 2022;13:937703. 35959407 10.3389/fneur.2022.937703 PMC9361122

[15] Fiorino F Pizzini FB Beltramello A Barbieri F. Progression of endolymphatic hydrops in Ménière’s disease as evaluated by magnetic resonance imaging. Otol Neurotol. 2011;32:1152–7. 21817938 10.1097/MAO.0b013e31822a1ce2

[16] Sone M Yoshida T Sugimoto S Kobayashi M Teranishi M Naganawa S. Pathological significance and classification of endolymphatic hydrops in otological disorders. Nagoya J Med Sci. 2022;84:497–505. 36237884 10.18999/nagjms.84.3.497 PMC9529623

[17] Domínguez P Manrique-Huarte R Suárez-Vega V López-Laguna N Guajardo C Pérez-Fernández N. Endolymphatic hydrops in fluctuating hearing loss and recurrent vertigo. Front Surg. 2021;8:673847. 34136529 10.3389/fsurg.2021.673847 PMC8202684

[18] de Pont LMH van Steekelenburg JM Verhagen TO. Hydropic ear disease: correlation between audiovestibular symptoms, endolymphatic hydrops and blood-labyrinth barrier impairment. Front Surg. 2021;8:758947. 34805261 10.3389/fsurg.2021.758947 PMC8601159

[19] Xie W Shu T Liu J. The relationship between clinical characteristics and magnetic resonance imaging results of Meniere disease: a prospective study. Sci Rep. 2021;11:7212. 33785791 10.1038/s41598-021-86589-1 PMC8010013

[20] Maeda Y Kojima K Takao S Omichi R Kariya S Ando M. Endolymphatic hydrops on magnetic resonance imaging may be an independent finding on aging in neurotologic patients. Otol Neurotol. 2023;44:737–41. 37400262 10.1097/MAO.0000000000003945

[21] Dieterich M Hergenroeder T Boegle R. Endolymphatic space is age-dependent. J Neurol. 2023;270:71–81. 36197569 10.1007/s00415-022-11400-8 PMC9813103

[22] Park CJ Cho YS Chung MJ. A fully automated analytic system for measuring endolymphatic hydrops ratios in patients with Meniere disease via magnetic resonance imaging: deep learning model development study. J Med Internet Res. 2021;23:e29678. 34546181 10.2196/29678 PMC8493456

[23] Cho YS Cho K Park CJ. Automated measurement of hydrops ratio from MRI in patients with Meniere’s disease using CNN-based segmentation. Sci Rep. 2020;10:7003. 32332804 10.1038/s41598-020-63887-8 PMC7181627

[24] van der Lubbe MFJA Vaidyanathan A de Wit M. A non-invasive, automated diagnosis of Meniere’s disease using radiomics and machine learning on conventional magnetic resonance imaging: a multicentric, case-controlled feasibility study. Radiol Med. 2022;127:72–82. 34822101 10.1007/s11547-021-01425-w PMC8795017

